# A Large Intrathoracic Hiatal Hernia as a Cause of Complete Heart Block

**DOI:** 10.1155/2021/6697016

**Published:** 2021-07-09

**Authors:** Ali Abbood, Hareer Al Salihi, Jorge Parellada, Mario Madruga, S. J. Carlan

**Affiliations:** Department of Internal Medicine, Division of Academic Affairs and Research, Orlando Regional Healthcare, Orlando, Florida, USA

## Abstract

Hiatal hernia is a not uncommon anatomic disorder resulting in portions of the bowel occupying space in the thoracic cavity. There are a number of antecedent risk factors including obesity but not hiatal hernias resulting in symptoms. When symptoms do occur, they can include chest pain, nausea, abdominal pain, and gastroesophageal reflux. Cardiac arrhythmias have also been reported as associated conditions resulting from a hiatal hernia. To date, however, a complete heart block secondary to a hiatal hernia has not been reported. An 88-year-old female with a history of GERD (gastroesophageal reflux disease) was found to have a large hiatal hernia at endoscopy after she presented to the emergency department with nausea and abdominal pain. Prior to her scheduled surgical repair, she developed symptomatic third degree heart block which resolved with nasogastric tube deflation of the gastric contents. After surgical repair of the hiatal hernia, she developed episodes of atrial fibrillation with rapid ventricular response and was started on diltiazem. She eventually converted back to normal sinus rhythm and remained dysrhythmia free. In addition to other known arrhythmias associated with hiatal hernia, a complete heart block can also be seen. Acute management requires deflation of the chest occupying hernia. This appears to be the one of the first reported cases of complete heart block caused by hiatal hernia.

## 1. Introduction

Hiatal hernia is a common pathology which increases in incidence with age. It is associated with (GERD) gastroesophageal reflux disease, a widespread condition that may present as atypical chest pain. Cases of patients with large hiatal hernia causing electrocardiographic changes have been well documented, manifesting as atrial fibrillation [1], atrial flutter [2], ST segment elevation [3], and others, but to date, there have been no reports of complete heart block caused directly by hiatal hernia. Here, we discuss one of the first reported documented cases of a hiatal hernia causing complete heart block.

## 2. Case Presentation

**Figure 1:**
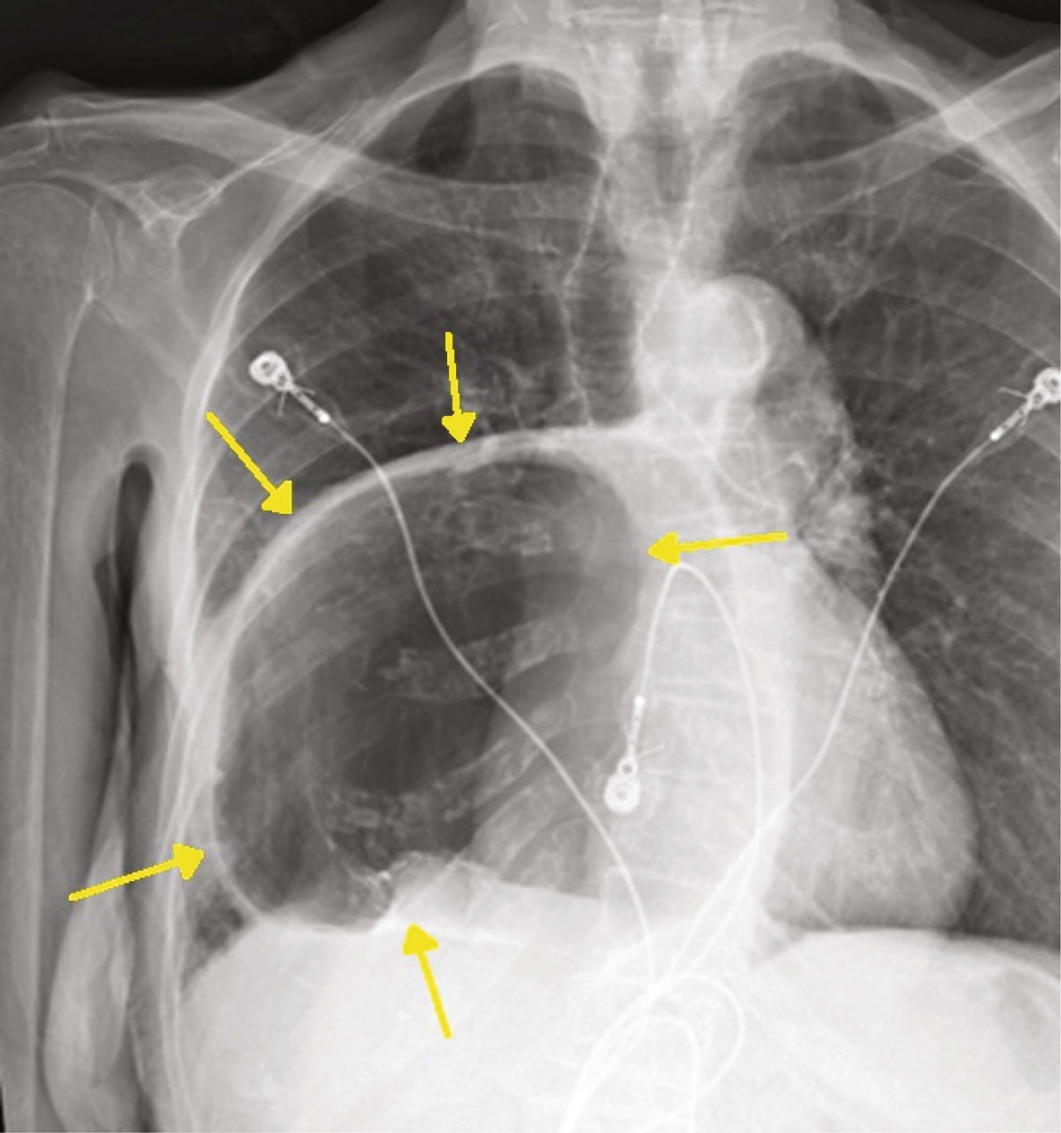
Upright chest X-ray showing large intrathoracic hiatal hernia between the yellow arrows.

**Figure 2:**
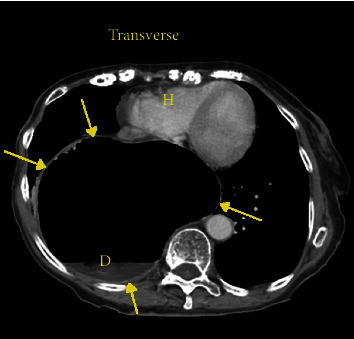
Transverse chest CT of the abdomen showing large hiatal hernia with concern for gastric volvulus or gastric outlet obstruction. The large hernia is between the yellow arrows. The heart is “H,” and the debris in the bowel is “D.”

**Figure 3:**
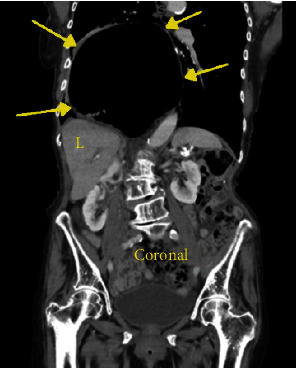
Coronal CT of chest and abdomen showing the large hiatal hernia located above the diaphragm with the liver “L” apparent.

**Figure 4:**
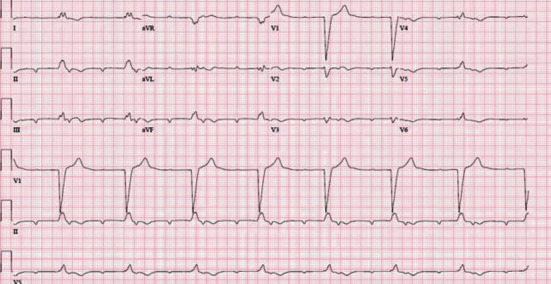
12-lead EKG showing complete heart block.

**Figure 5:**
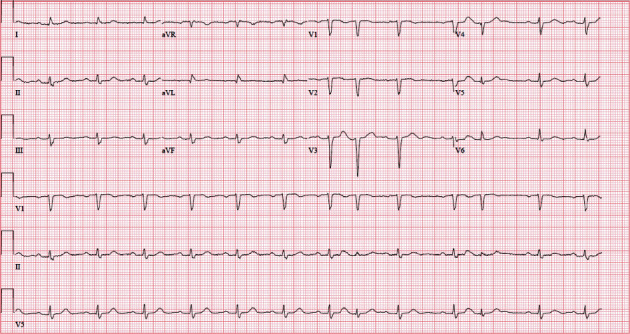
12-lead EKG obtained after NG tube placement and patient reverting back to normal sinus rhythm.

An 88-year-old female with a past medical history of gastroesophageal reflux disease, hypertension, and anxiety presented to the emergency department with nausea and abdominal pain after chest X-ray and CT (computed tomography) scan of the abdomen performed at an outside facility showed a large hiatal hernia with possible volvulus or gastric obstruction (Figures 1–3) at an outside facility. She had no past history of known treated cardiac dysrhythmia. Her vital signs were normal, and physical exam was only remarkable for epigastric abdominal pain. The patient was admitted and evaluated with GI series barium study, which showed a thoracic stomach with gastric obstruction. An EGD (esophagogastroduodenoscopy) was performed which confirmed the presence of a large hiatal hernia. Surgical intervention was planned to correct the anatomical defect, but the day prior to her planned surgery, she ate a large meal and became hypotensive, with her 12-lead ECG showing complete heart block (Figure 4). The patient was promptly sent to the intensive care unit (ICU) for monitoring. She had a nasogastric tube inserted for decompression, which converted the complete heart block back to normal sinus rhythm (Figure 5). Three days later, she underwent successful laparoscopic robotic-assisted hiatal hernia repair and gastrostomy tube placement. Postoperatively, the patient had no recurrence of complete heart block, and she was transferred from the ICU to the cardiac unit. There, she proceeded to experience episodes of atrial fibrillation with rapid ventricular response. Echocardiography was performed and was significant for an ejection fraction of 65-69%, moderate aortic stenosis, and pulmonary hypertension. The patient was placed on diltiazem and eventually converted back to normal sinus rhythm. She was discharged to a skilled nursing facility with diltiazem and apixaban and was instructed to follow up with cardiology and thoracic surgery as an outpatient.

## 3. Discussion

Hiatal hernia is defined as an abnormal protrusion of the stomach above the diaphragm from the esophageal hiatus. While hiatal hernias most often only involve protrusion of the stomach as the protruding organ, it may also contain the transverse colon, small bowel, spleen, or some combination of these. Risk factors for hiatal hernias include obesity, age-related changes in the diaphragm, and increased intra-abdominal pressure due to conditions like chronic coughing, constipation, exercising, and lifting heavy objects. Hiatal hernias may be asymptomatic or may present with gastroesophageal reflux disease, dysphagia, odynophagia, hoarseness, or asthma. Extreme cases may cause compression of the heart and pulmonary veins, leading to a presentation of pulmonary edema and cardiac failure [4]. Atrioventricular (AV) block is a cardiac electrical disorder defined as impaired (delayed or absent) conduction from the atria to the ventricles. The severity of the conduction abnormality is described in degrees: first-degree, second-degree, type I (Wenckebach or Mobitz I) or type II (Mobitz II), and third-degree (complete) AV block [5].

Large hiatal hernias can induce electrocardiographic changes, and atrial tachycardia, atrial fibrillation, supraventricular tachycardia, atrial flutter, and T wave changes have all been previously reported, but the exact mechanism of such arrhythmias is not well understood. One current hypothesis suggests that it is the increase in the direct or indirect pressure on the global surface of the heart by the hernia which causes these electrical changes seen on EKG [6]. Another suggests that it is actually due to the compression on the heart by the thoracic stomach causing anatomic conduction block or disruption in the vagal nerve [7]. Intense vagal stimulation has been described before to causing syncope and bradycardia in “swallow syncope syndrome.” The pathophysiologic explanation is due to the common innervation of the esophagus and heart by the vagus nerve. The mechanoreceptors in the esophagus sense the distension and send signals along the esophageal plexus vagus nerve to the brainstem and then efferent impulses from the brainstem reach the sinoatrial (SA) node and the AV node, that leads to developing various bradyarrhythmias and temporary reduction of the cardiac output, which in result leads to cerebral hypoperfusion and syncope [8].

The presented patient had a huge hiatal hernia, and the food intake was the trigger for her hypotension and telemetry at that time it showed complete heart block. After the patient had a successful laparoscopic surgery and hiatal hernia repair, she did not develop any recurrent AV blocks. She did have a brief atrial fibrillation episode that converted back to normal sinus rhythm, which can be a postoperative episode as the patient has no history of AFib or palpitations before. Our patient was monitored on telemetry for days prior to discharge, and no recurrent events were recorded. She followed with cardiology outpatient and was on Holter monitor for 2 weeks which showed no significant events and leads to the conclusion that the surgical repair to the hernia resulted in complete resolution of the AV block.

We hypothesize that the hernia has caused a mechanical conduction block on the AV node or an increase in the firing of the vagal nerve by the mechanism mentioned above.

## 4. Conclusion

We present a rare case of a large hiatal hernia causing complete heart block and symptomatic hypotension resolving with nasogastric tube placement. To our knowledge, this appears to be one of the first cases to report these findings. We performed a MEDLINE search of the English language literature via PubMed from January 1, 1946 to the present, using the MeSH terms and keywords “hiatal hernia,” “heart block,” and “atrioventricular block.” We could find, however, no cases of complete heart block associated with hiatal hernia. We concluded that the symptomatology of the patient was related to compressive effects caused by the hernia.

## Data Availability

The clinical and treatment data used to support the findings of this study are included within the article.
